# Metastatic high-grade meningioma: A case report and review of risk factors for metastasis

**DOI:** 10.1093/noajnl/vdad014

**Published:** 2023-03-01

**Authors:** Destiny D Bailey, Eric Y Montgomery, Tomas Garzon-Muvdi

**Affiliations:** Department of Neurosurgery, University of Texas Southwestern Medical Center, School of Medicine, Dallas, Texas, USA; Department of Neurosurgery, University of Texas Southwestern Medical Center, School of Medicine, Dallas, Texas, USA; Department of Neurosurgery, Emory University, Atlanta, Georgia, USA

**Keywords:** extracranial metastasis, meningioma, metastatic, recurrent meningioma

**Meningiomas are the most common primary tumor of the CNS, and although they usually have a good prognosis, meningiomas can metastasize to extracranial sites, greatly worsening the prognosis. Due to its infrequency, the clinical and molecular risk factors associated with this event are poorly characterized in the literature. A 69-year-old man was found to have a meningioma in the left parieto-occipital parafalcine region. Over the subsequent decade, the patient experienced multiple local tumor recurrences, including invasion through the scalp and superior sagittal sinus. The tumor was WHO grade 2–3 and next-generation sequencing was notable for TERT promoter mutation, homozygous deletion of CDKN2A, and high variant allele frequency of NF2. The tumor metastasized to the axial and appendicular skeleton, liver, kidney, and lung, and the patient ultimately succumbed to disease. In summary, this is the first report of multiple simultaneous and anatomically distinct extracranial metastases from a primary intracranial meningioma.**

Meningiomas constitute over 30% of all primary Central Nervous System (CNS) tumors.^1,2^ Extracranial metastasis is a rare complication of what is a relatively common and well-treated primary tumor. The best currently available evidence estimates have the rate of metastasis at less than 1% in all meningioma patients. However, when metastasis does occur, it reduces survival from 88.3% (95% Confidence Interval (CI): 88.1%–88.5%) at 5 years to 66.5% (95% CI: 64.2%–68.8%).^3,4^ Watershed advances in our understanding of tumor biology have also provided a new depth of understanding for the prognostic implications of the tumor’s molecular state. The most recent update to the WHO classification of CNS tumors highlights molecular prognostic indicators such as mutations in *SMARCE1*, *BAP1*, *KLF4*/*TRAF7*, or *TERT* promoter, deletion of *CDKN2A*/*B*, and methylation profiling.^5^ Preclinically, Youngblood et al. recently found that mutations in the Hedgehog signaling pathway were an independent predictor of worsen progression-free survival.^6,7^ Patel et al. discovered that the loss of DREAM complex tumor suppressor function was required for tumor recurrence.^8^ Herein, we describe a case of metastatic meningioma with multifocal metastases after multiply recurrent disease.

## Detailed Case Description

The disease course began when a 69-year-old patient presented with a seizure to a hospital following a motor vehicle accident in December 2011, at which time he was diagnosed with a “benign” meningioma. The tumor measured 2.5 × 6.0 × 5.0 cm, without midline shift and only minimal mass effect into the left lateral ventricle. Unfortunately, images from the outside institution were unable to be viewed. After undergoing an uncomplicated left posterior parieto-occipital craniotomy for resection of the meningioma, the patient developed a postoperative left parieto-occipital venous stroke leading to hemorrhage and resulting in subsequent right-sided hemiparesis, right homonymous hemianopsia, and aphasia. Following this incident, he continued to have intractable seizures despite multiple antiepileptic drug trials. Of note, following one week of Dilantin, the patient developed a rash, fever, and mucus membrane swelling, diagnosed as Drug Reaction with Eosinophilia and Systemic Symptoms Syndrome.

In February of 2012, the now 70-year-old patient presented for the first time to the University of Texas Southwestern Medical Center (UTSW) with continued intractable seizures, resulting in a month-long stay in the hospital. Shortly after discharge, he presented back to the UTSW emergency department (ED) with altered mental status (AMS) secondary to bilateral pulmonary emboli, deep vein thrombosis (DVT) in the right femoral artery, and an acute infarct in the right cerebellum, suspected to be cardioembolic. Although he continued to have left central parietal focal motor seizures and left central parietal cerebral dysfunction, his condition began to improve over the next several months and years.

**Figure 1.**
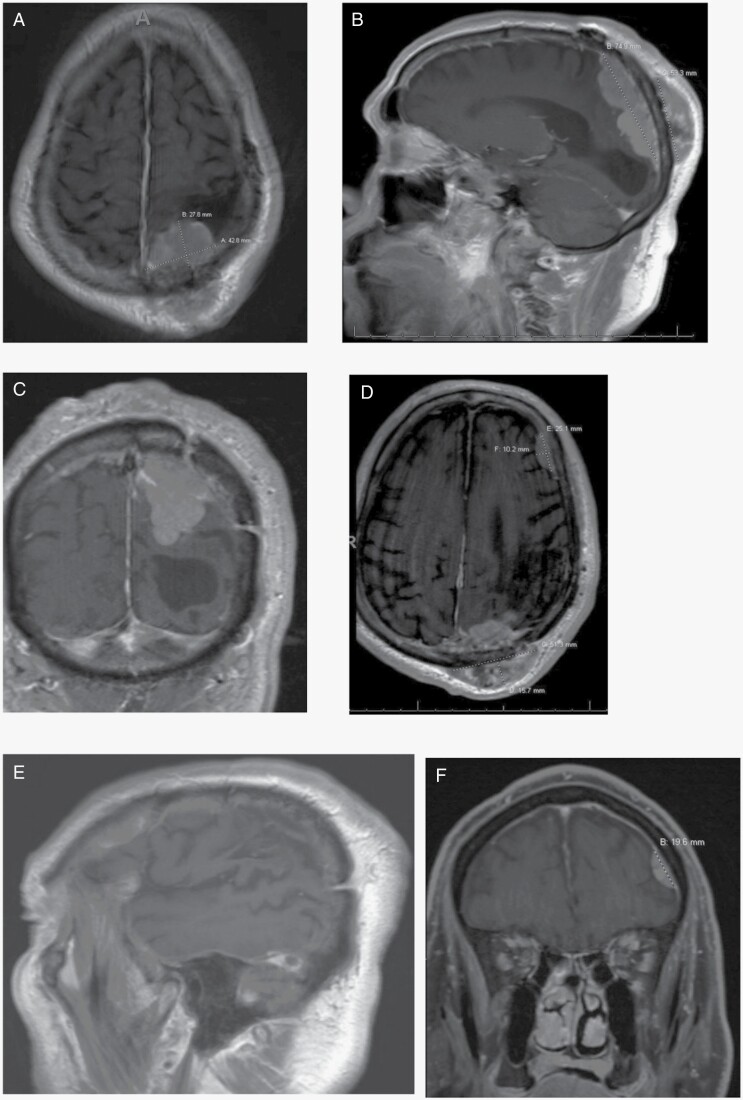
(**A**) MR AX T1 Flair on 3/13/2020 showing multilobulated mass along left parietal/occipital lobes and extracranial mass. (**B**) MR SAG T1 Flair on 3/13/2020 showing multilobulated mass along left parietal/occipital lobes and extracranial mass. (**C**) MR COR T1 Flair on 3/13/2020 showing multilobulated mass along left parietal/occipital lobes. (**D**) MR AX T1 Flair post-contrast on 03/13/2020 shows new frontal lesion. (**E**) MR SAG T1 Flair post-contrast on 03/13/2020 shows new frontal lesion.

With the exception of continued visits to the ED for hypertensive emergencies, AMS, and partial-complex and focal motor seizures, the patient’s condition regarding the meningioma was presumably unchanged until February 2020 when he presented to his primary care physician at 78-years-old with a “new lump on his head,” adjacent to the 2011 craniotomy site. Computed tomography (CT) revealed a large extracranial mass adjacent to the left parietal craniotomy, with an intracranial component located along the left parietal and occipital lobes. Magnetic resonance (MR) imaging identified the new, multilobulated mass as consistent with meningioma, with broad-based dural attachment (Figure 1A–C). The tumor extended through the bone into the subgaleal space, measuring 4.3 × 2.4 × 7.1 cm intracranially and extending extracranially at 5.4 × 1.5 × 4.7 cm. MRI additionally revealed a second, smaller mass along the left frontal convexity (Figure 1D–F), measuring 2.5 × 1.0 × 2.0 cm.

**Figure 2.**
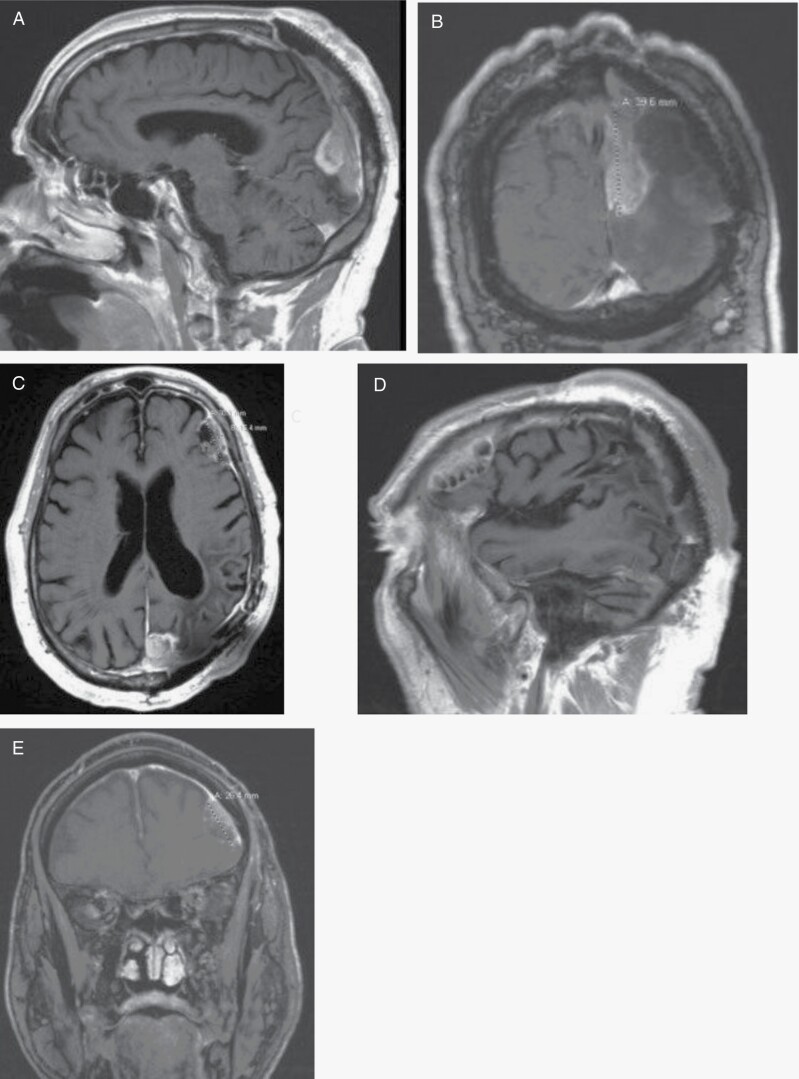
(**A**) MR SAG T1 Flair post-contrast on 10/10/2020 shows the parafalcine lesion. (**B**) MR COR T1 Flair post-contrast on 10/10/2020 shows growth of parafalcine lesion and invasion into superior sagittal sinus. (**C**) MR AX T1 Flair post-contrast on 10/10/2020 shows the parafalcine and frontal lesion. (**D**) MR SAG T1 Flair post-contrast on 10/10/2020 shows the increase in size of the frontal lesion. (**E**) MR COR T1 Flair post-contrast on 10/10/2020 shows the increase in size of the frontal lesion.

In March 2020, the patient underwent an uncomplicated second left parieto-occipital craniotomy (& a titanium mesh cranioplasty) to resect the new left parietal mass, although the smaller mass along the left frontal convexity was untouched. Postsurgical MRI imaging of the resection site showed a small residual left parafalcine component along the posterior interhemispheric fissure. The patient was discharged following surgery to in-patient rehab without any new deficits. Pathological analysis resulted in WHO grade 2–3 atypical meningioma. Despite 2 sessions of Gamma Knife Radiation Surgery to the small frontal lesion and residual left parafalcine lesion, by the end of 2020 the left parafalcine lesion invaded the superior sagittal sinus (SSS), and the frontal lesion increased in size (Figure 2A–E).

Although the size of the frontal lesion decreased by February 2021, MRI revealed increased SSS invasion, a new subcutaneous nodule, and many others newly extending into the left temporal dura. Due to the dramatic progression of disease, the patient underwent a third left parieto-occipital craniotomy in February 2021 to resect the recurrent meningioma extending through the bone. Pathology revealed a recurrent anaplastic WHO grade 3 meningioma. Next-generation sequencing was significant for the TERT promoter mutation, homozygous deletion of CDKN2A, and high variant allele frequency of NF2. Following surgery, the patient began fractionated radiation and chemotherapy consisting of daily everolimus and monthly octreotide.

**Figure 3.**
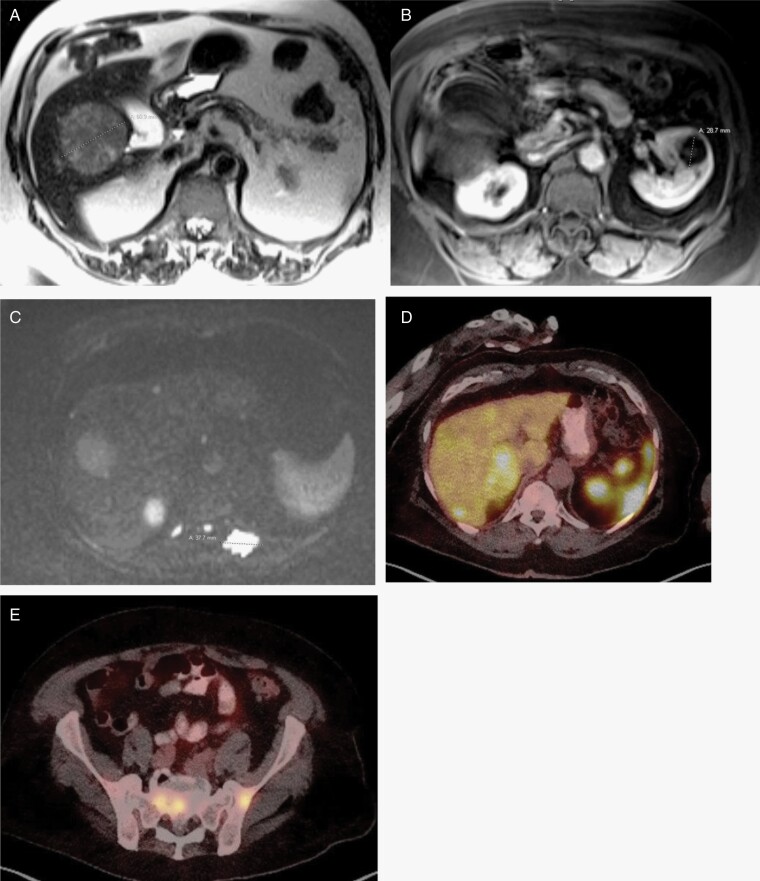
(**A**) MR on 09/08/2021 shows liver metastasis. (**B**) MR on 09/08/2021 shows kidney metastasis. (**C**) MR on 09/08/2021 shows spinal metastasis. (**D**) DOTATE PET on 09/10/2021 shows liver metastases. (**E**) DOTATE PET on 09/10/2021 shows sacral and vertebral metastases.

Unfortunately, MRI imaging in July 2021 showed increased interval progression of disease in the brain. Chemotherapy was stopped and Avastin (bevacizumab) monotherapy infusions were started every 2 weeks at 10 mg/kg. Just 2 months later, in September 2021, the now 79-year-old patient returned to the ED cachectic, complaining of weight loss, decreased appetite, abdominal fullness, and epigastric pain for 1 week. MRI confirmed metastatic lesions in the liver (Figure 3A), left kidney (Figure 3B), and vertebral skeleton (Figure 3C), as well as corresponding DOTATE positron emission tomography (PET) scans (Figure 3D and E). Hypometabolic lesions found in the lower left lobe of the lung and left kidney did not have significant DOTATE uptake and were ultimately thought to be either metastatic or due to primary malignancy. CT-guided biopsy of the liver revealed the 4.0 cm hepatic lesion to be metastatic, atypical meningioma with morphological features similar to prior lesions resected from the brain.

In light of the metastatic findings, Avastin monotherapy was stopped and chemotherapy consisting of everolimus and octreotide was resumed. The patient was immediately referred to nuclear medicine/diagnostic radiology and started on Lutathera (177 Lu-DOTATE) every 2 months for a total of 4 infusions, which were completed at the end of April 2022. Apart from developing anemia, thought to be a side effect of Lutathera, the patient responded well to the treatment. The neuro-oncology team reported that the patient looked physically improved following the Lutathera treatments from his initial presentation of liver metastasis at the end of 2021.

In June 2022, MRI imaging showed continued worsening on intra- and extracranial lesions with associated mass effect, but no definite midline shift. One month later, a PET scan revealed suspicion for progression of metastatic disease of extracranial and liver lesions. However, multiple new and increased DOTATE lesions were found bones of the axial and appendicular skeleton. The day following his last PET scan in July 2022, the patient presented to the ED and was admitted with anxiety, headaches, worsening gait imbalance, and AMS. Long-term EEG revealed subclinical seizures arising from the left frontal lobe, for which Valproic Acid (500 mg BID) was added to his regimen, in addition to his regular Vimpat (200 mg BID) and Keppra (2g BID). After 2 days in hospital, the patient’s mental status improved with this regimen. Of note, the patient was also consulted by cardiology with incidental findings of bilateral lower extremity edema and severe mitral valve regurgitation, for which he was medically treated without surgical intervention.

Ultimately, the role of radiation treatment for the patient’s brain and metastatic disease progression was determined to have low utility. The radiation-oncology team deferred whole brain radiation for tumor and seizure control due to the anaplastic tumor histology and insignificant response to prior gamma knife radiation treatments. Palliative radiation to distant sites was also deferred due to preserved liver function and lack of bony pain. Considering the lack of remaining viable treatment options, the patient and his family opted to consult at-home hospice agencies. Two weeks after discharge, the patient passed away in his home. The official cause of death is unknown.

## Discussion

Meningiomas comprise 38.3% of all primary CNS tumors,^1^ and generally carry a favorable prognosis with a 5-year survival rate of 88.0%. However, the occurrence of metastasis reduces the 5-year survival to 66.5%.^2^ Due to the rarity, there are few large series on metastatic meningiomas. Dalle Ore et al. recently performed the largest such study and found that the overall incidence of extracranial metastasis was 0.67%.^3^ The dearth of literature leaves risk factors for metastasis poorly understood. The goal of this study is to highlight a complex case of meningioma metastasis in comparison to the known clinical and molecular risk factors for this rare occurrence. To the authors’ knowledge, this is the first report of a primary intracranial meningioma with extracranial metastases simultaneously involving multiple distinct sites, including the kidney and thoracic spine.

The described patient had multiple risk factors for metastasis, as highlighted in the literature review below. First, and perhaps most expected, Dalle Ore et al. and Garzon et al. both demonstrated that higher grade tumors have a greater propensity for metastasis.^3,9–12^ Similarly, recurrence of the primary tumor occurred in the majority of metastatic patients, despite being a rare occurrence overall.^3,4^ Location-wise, non-skull base tumors have been shown to have a higher propensity for metastasis, where proximity may facilitate invasion of the venous sinuses and scalp.^9,12,13^ The subject of this case report demonstrated scalp invasion, and this risk has been echoed by a recent case report and described by 2 previous case series.^14–16^ Garzon et al. also proposed that cranioplasty for these patients may serve as a nidus of invasion into the scalp and suggested the utilization of nonporous material when indicated.

Literature review of the currently available case reports yields 42 cases, with common themes reflecting the proposed risk factors above.^17^ The lung is the most common site of metastasis with 19 such reports, followed by bone with 12. Vertebral body metastases were included in this value, while intradural metastases were not. Other noted sites of metastasis include the liver,^18–22^ adrenal gland,^23^ and pleura.^24–28^ In rare instances, metastases have been reported in other sites such as the sternum, clavicle, or ribs.^29–32^ This patient’s metastases involved multiple common sites, such as the lungs and liver, as well as multiple rarer sites. Most vertebral body metastases have been reported in the cervical spine, with only one previous report of thoracic metastases.^33^ As far as the authors are aware, this is the first report of meningioma metastases to the kidney. Similarly, there are few, if any, reports of multiple simultaneous extracranial metastases involving distinct organs and tissues.

A recent technological advancement that may improve detection of meningioma metastases is ^68^Ga-DOTATATE PET imaging. Taking advantage of the well-described association between meningiomas and somatostatin receptor 2 (SSTR2), ^68^Ga-DOTATATE uses an SSTR2 analog to enable distinguishment of postoperative and postradiation changes from meningioma tumor tissue.^34–37^ In 2015, Rachinger et al. demonstrated that ^68^Ga-DOTATATE PET imaging had a significantly higher sensitivity than the gold standard of contrast-enhanced MRI (CE-MRI) in detecting primary and recurrent meningioma tissue, regardless of WHO grade.^34^

Lastly, recent technological advances in molecular biology have allowed for a new depth of understanding for meningioma biology. The highlighted patient had an atypical primary meningioma per WHO grading (at the most recent relapse), as well as homozygous CDKN2A deletion, NF2 high variant allele frequency, and TERT promoter mutation that similarly corroborate the described risk factors in the literature.^8,38–41^ Although few, if any, studies have directly analyzed metastatic meningiomas, key implications can be inferred from the risk factors associated with tumor progression. NF2 mutant meningiomas are more likely to be characterized by atypical histology and genetic instability^38,42,43^ and have been identified in multiple case reports of meningioma metastasis.^44,45^ Recent genomic studies have demonstrated that meningiomas with mutations in *NF2* were more likely to recur than those with *KLF4*, *POLR2A*, or *SMARCB1* mutations.^6,7^ Lastly, methylation profiling has been shown to be a stronger indicator of recurrence risk than classic prognosticators such as WHO grade.^46^

## Conclusion

The current case report highlights the contemporary understanding of clinical and molecular risk factors for metastasis from a primary intracranial meningioma. The authors hope that the corresponding review will serve as a template for clinical and preclinical studies moving forward in order to better understand how the described risk factors impact the incidence of metastasis as well as survival outcomes.
